# Removal of Toxic Mercury from Petroleum Oil by Newly Synthesized Molecularly-Imprinted Polymer

**DOI:** 10.3390/ijms160510562

**Published:** 2015-05-08

**Authors:** Nor Ain Shahera Khairi, Nor Azah Yusof, Abdul Halim Abdullah, Faruq Mohammad

**Affiliations:** 1 Department of Chemistry, Faculty of Science, Universiti Putra Malaysia, 43400 Serdang, Selangor, Malaysia; E-Mails: khairinorainshahera@yahoo.com (N.A.S.K.); halim@upm.edu.my (A.H.A.); 2 Institute of Advanced Technology, Universiti Putra Malaysia, 43400 Serdang, Selangor, Malaysia

**Keywords:** molecularly-imprinted polymer, cysteine complex, mercury removal, petroleum oil, Freundlich isotherm

## Abstract

In recent years, molecularly-imprinted polymers (MIPs) have attracted the attention of several researchers due to their capability for molecular recognition, easiness of preparation, stability and cost-effective production. By taking advantage of these facts, Hg(II) imprinted and non-imprinted copolymers were prepared by polymerizing mercury nitrate stock solution (or without it) with methacrylic acid (MAA), 2-hydroxyl ethyl methacrylate (HEMA), methanol and ethylene glycol dimethacrylate (EGDMA) as the monomer, co-monomer solvent (porogen) and cross-linker, respectively. Thus, the formed Hg(II) imprinted polymer was characterized by using Fourier transform infrared spectroscopy (FTIR), field emission scanning electron microscopy (FESEM), Brunauer, Emmett and Teller (BET) and thermal gravimetric analysis (TGA). The separation and preconcentration characteristics of Hg(II) imprinted polymer were investigated by solid phase extraction (SPE) procedures, and an optimal pH of 7 was investigated as ideal. The specific surface area of the Hg(II) imprinted polymer was found to be 19.45 m^2^/g with a size range from 100 to 140 µm in diameter. The maximum adsorption capacity was observed to be 1.11 mg/g of Hg(II) imprinted beads with 87.54% removal of Hg(II) ions within the first 5 min. The results of the study therefore confirm that the Hg(II) imprinted polymer can be used multiple times without significantly losing its adsorption capacity.

## 1. Introduction

Mercury (Hg) is classified as one of the most toxic heavy metals on Earth, where it can be adsorbed through the skin, oral routes or by inhalation and exhibit adverse reactions on human health [1]. This further leads to a variety of health defects, including neurological, renal, respiratory, immune, dermatologic, reproductive and developmental neurotoxicity [2]. According to WHO guidelines, the mercury limitation is restricted to 1 µg/L in water for total mercury and 1 µg/m^3^ in air. In addition, the WHO estimated a tolerable concentration of 0.2 µg/m^3^ for long-term inhalation exposure to elemental mercury vapor and confined for a tolerable intake of 2 µg/kg body weight per day [3]. Furthermore, from the understanding of the toxicity effects, mercury and its compounds are listed in almost all classes of priority pollutants, where different guidelines and regulations have been set on limiting their levels in water, soil, sediments and in the environment [4].

According to the U.S. EPA 1997, combusted hydrocarbon was classified as one of the major anthropogenic sources of mercury emissions to the atmosphere in the U.S. Besides this, aquatic mercury contamination is caused by liquid discharges from petroleum refineries and petrochemical plants surrounding the rivers, where mercury is identified to be present as one of the most common heavy metals in petroleum oil [5]. In many forms of crude oils, a variety of mercury-containing species are found to be present, including elemental mercury, Hg(I) compounds, Hg(II) compounds and a combination thereof [6]. The presence of these elements can cause detrimental effects, as they pose significant product quality, environmental and safety issues. Furthermore, the consequences of mercury in feeds on processing systems includes the degradation of equipment parts, poisoning of catalysts, toxic waste generation and increased risk of the health and safety of workers. All of these factors can directly or indirectly be responsible for a reduction in the quality of the final hydrocarbon products [7].

Chemical adsorption, gas stripping and chemical precipitation methods are currently being used for removing mercury from crudes and other hydrocarbon liquids prior to their processing in order to avoid the problems of poisoning. Mercury removal with sorbent beds is a method used to scavenge the mercury from gas and liquid hydrocarbon streams. Gas phase treatment systems primarily consist of sulfur impregnated carbon, metal sulfide on carbon or alumina and regenerative molecular sieves. As for the hydrocarbon liquid streams, the systems consist of iodide-impregnated carbon [8], metal sulfide on carbon or alumina, a mol-sieve amalgam system and a two-step processing consisting of hydrogenation conversion catalyst followed by metal sulfide reaction with elemental mercury. All of the commercialized methods have both advantages, as well as disadvantages, depending on the feed composition and stream location. For example, mol-sieve amalgamation sorbents do not operate at high efficiencies if the organic form of mercury is present in a significant concentration. Other than that, sulfur-impregnated carbon is soluble in liquid hydrocarbon and cannot be used in process locations [9,10].

In recent years, the molecular imprinting technique has attracted considerable interest in many areas of science, such as in physics, chemistry, biochemistry and biotechnology, owing to its high degree of selectivity and affinity towards the target molecules [11]. The molecularly-imprinted polymer (MIP) processed by the molecular imprinting technique can be described as the synthetic polymeric materials having specific recognition sites that are complementary in shape, size and functional groups with regards to the template molecule [12]. This unique ability can recognize the template molecule used in the imprinting process, even in the presence of compounds having a similar structure and functionality to the template [13]. The MIPs tend to be simple and inexpensive to prepare. The MIPs also offer several advantages, including high mechanical strength, resistance to elevated temperatures and pressure and stability in the presence of extreme acidic, basic, metal ionic and organic solvent conditions [14].

Based on the selection of features and advantages offered by the molecular imprinting technique, the aim of the present work is to introduce a suitable MIP probe that can be useful for the selective trapping of mercury and its compounds from a mercury removal system. For that, the synthesis method we used involves free radical or chain growth polymerization, as these are the most commonly applied methods in industries for the conversion of monomers into polymer. We have selected cysteine monomer to form a polymerized complex in the presence of other organic molecules, and the reason for selecting cysteine is that it contains some electronegative groups, such as –NH_2_, –SH and –COOH, in its structure. These groups with their lone pair of electrons can easily form the electrostatic and van der Waals forces of attractions with the electropositive Hg compounds, and in this way, the adsorption can be easier, stronger and stable without any leaching [15]. Thus, synthesized MIP was tested for its mercury trapping capacity against various parameters, including the pH, dosage, sorption kinetics, sorption isotherms, reusability and selectivity. From the analysis of results and by consideration of the versatility, it is indicated that a high level of selectivity and recognition can be achieved with our MIP, which further confirms the future use of MIPs as a promising sorbent material in mercury removal systems.

## 2. Results and Discussion

### 2.1. Characterization Studies

#### 2.1.1. FTIR

**Figure 1:**
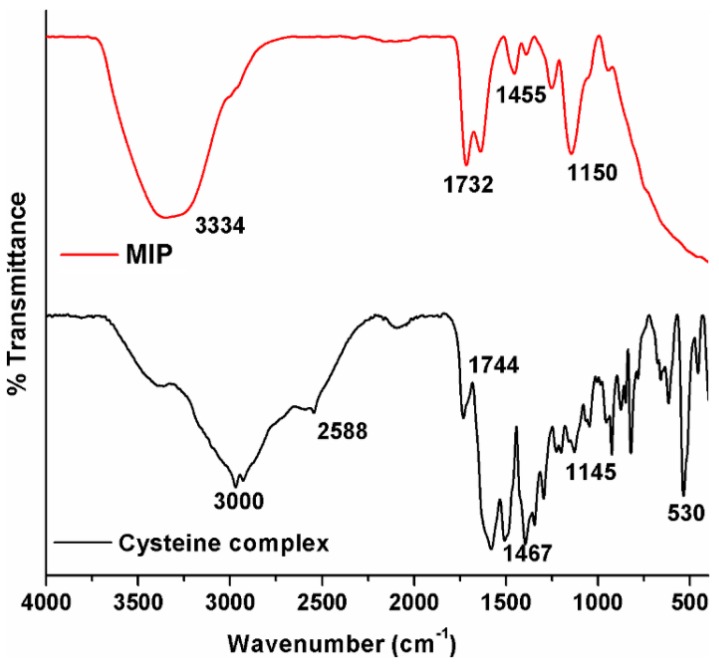
Comparison of FTIR spectra of the cysteine complex and the molecularly-imprinted polymer (MIP) Hg(II)-cysteine complex.

The Fourier transform infrared (FTIR) spectra recorded by making use of the universal attenuated total reflectance (UATR) method for the cysteine complex along with the Hg(II)-cysteine complex (MIP) are shown in Figure 1. From the figure, the spectrum of the cysteine complex showing the characteristic absorbance peak at 2588 cm^−1^ can be ascribed to –SH stretching vibration. It is obvious that there is no peak observed in the –SH region for MIP, indicating the absence of the –SH group in the polymer form. This confirms the complete polymerization of the –SH group in the cysteine complex with the Hg(II) template. The sulfhydryl groups can donate the lone pair of electrons to the empty orbit of metal ions alone. Similarly, the observation of a peak around 1744 cm^−1^ related to the –C=O group in the cysteine complex was found at 1732 cm^−1^ in MIP, confirming the persistence of the bonding without any further modifications. Furthermore, the observation of a sharp peak in the fingerprint region of around 1150 cm^−1^ in both samples can be attributed to the C–N and C–S stretching vibrations of the groups (–NH_2_ and –SH) available in cysteine. Further, the appearance and persistence of peaks in both the cysteine complex and MIP confirm the maintenance of the cysteine skeleton without much alteration, even in the MIP Hg(II)-cysteine complex [16].

#### 2.1.2. FE-SEM Analysis

**Figure 2:**
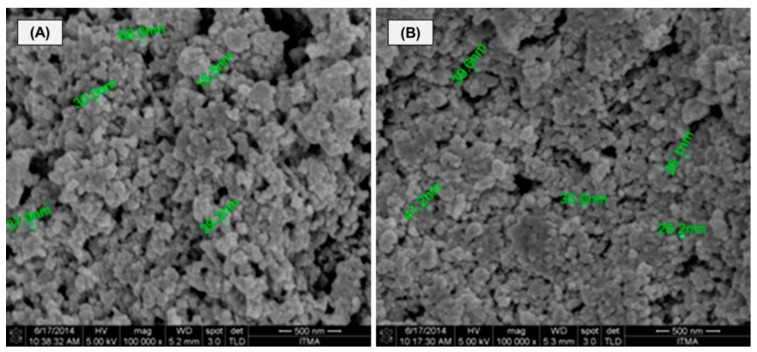
FE-SEM images of the Hg(II)-imprinted polymer (MIP) (**A**) and the non-imprinted polymer (NIP) (**B**) (magnification: 100,000×). Scale bar = 500 nm.

The structural morphology of MIP and non-imprinted polymer (NIP) beads is exemplified by the electron micrographs in Figure 2A,B. From the figure, all of the beads seem to possess a spherical shape, and the spots of MIP exhibit large particles packed together with a rough surface morphology (Figure 2A). However, the NIP morphology seems to contain densely packed particles with a smoother surface (Figure 2B), and this may be due to the fact that no specific binding sites have been created by the mercury template. Porosity is a very important factor, as it can change the surface area of the materials by creating new binding sites that are responsible for the selective removal of Hg(II) ions from solutions by means of trapping them [15].

#### 2.1.3. Thermal Stability

**Figure 3:**
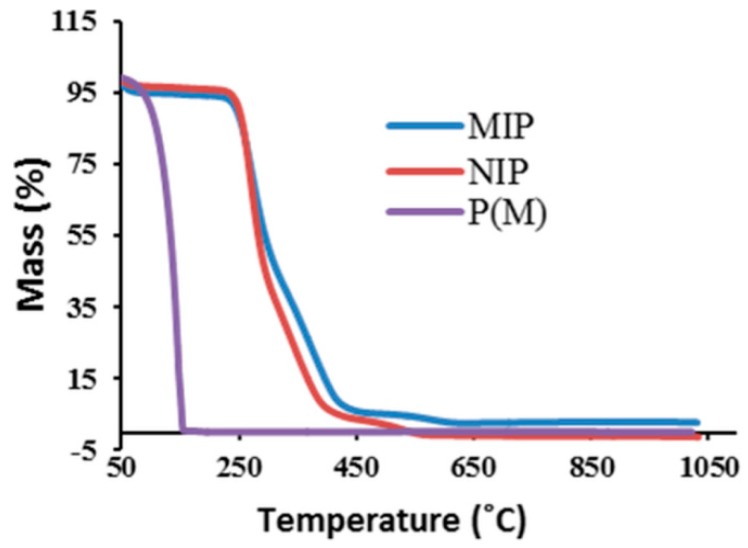
Studies of thermal gravimetric analysis (TGA) for the monomer (P(M)), molecularly-imprinted polymers (MIP), non-imprinted polymer (NIP).

The thermal analyses for the monomer, MIP and NIP were performed to understand the stability at various temperatures and to investigate the stages of decomposition (Figure 3). In the measurement process, the samples were heated from 35 to 1000 °C at a heating rate of 10 °C/min in a N_2_ atmosphere. From the figure, one can see a similar kind of degradation pattern between MIP and NIP. The monomer methacrylic acid (MAA) started to decompose at *T*_i_ of 145 °C, and the final decomposition *T*_f_ is observed at 197 °C. However, the initial compositions *T*_i_ of MIP and NIP are observed at 192 and 196 °C and final decompositions *T*_f_ at 671 and 601 °C, respectively. From the comparison of data of MIP and NIP against the monomer, it is indicated that the MIP and NIP have higher thermal stabilities due to the formation of a complex hybrid structure by means of polymerization. Within MIP and NIP, the final decomposition temperature of MIP is relatively higher than the corresponding NIP, indicating the stability of the MIP template at quite higher temperatures [17].

### 2.2. Effect of pH

**Figure 4:**
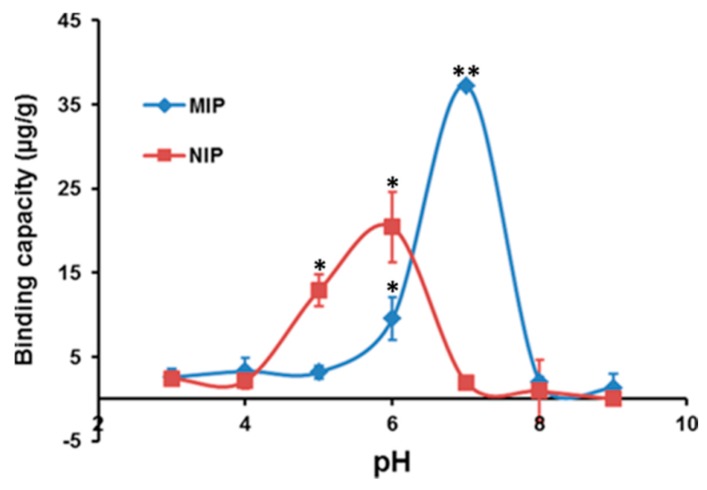
The effect of pH towards the sorption of Hg(II) for the MIP and NIP material (studies were carried out by taking 10 mg of MIP and NIP at a 25 °C). The results are expressed as the mean ± SD of the individual experiments; * and ** indicate the significance at *p <* 0.05 and *p <* 0.01* versus* the controls.

The effect of pH on Hg(II) sorption towards MIP and NIP was studied in the pH range from 3.0 to 9.0, and the results are presented in Figure 4. It can be seen from the figure that the binding capacity seems to be increased with an increase of pH for both materials. For the pH in the range of 3.0–4.0, the sorption was found to be low, owing to the high concentration and mobility of the H^+^ ions that get preferentially adsorbed onto the lone pairs of the MIP in comparison with Hg(II) ions [18]. Further, the sorption increased rapidly after pH 5.0, but the increasing rate for the NIP is slow compared to MIP. At a high pH value of >7.0, the binding capacity decreased due to the precipitation of the metal hydroxide and its quick decomposition further to oxide in both polymers [19]. Overall, the graph of NIP shows low binding capacity compared against the MIP due to the non-specific interaction between the Hg(II) ions and the polymer matrix. Furthermore, as seen from the figure, the optimum pH value for the sorption of Hg(II) from aqueous solution was obtained to be at pH 7.0, and therefore, the pH was adjusted to 7.0 for all of the subsequent studies.

### 2.3. Effect of Dosage on MIP

**Figure 5:**
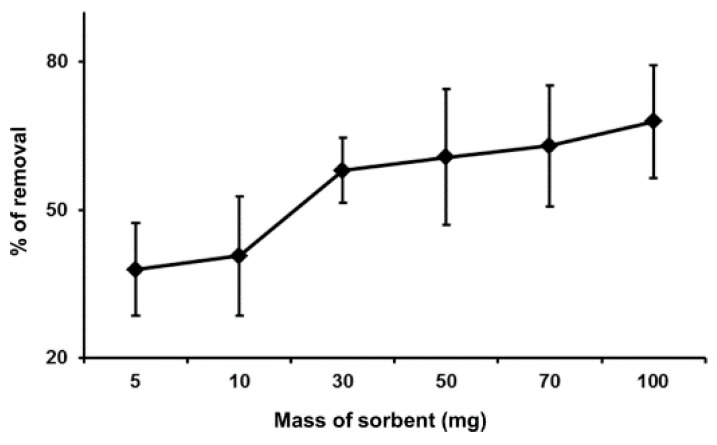
Effect of dosage on the percentage removal of Hg(II) (conditions: 10 mL of 200 ppb of Hg(II), at pH 7 and 25 °C, contact time of 10 s).

The dependence of the mass of sorbents (dosage) on the binding capacity of Hg(II) was studied by varying the amount of MIP from 5.0 to 100.0 mg, and the results are shown in Figure 5. From the figure, the graph shows that the percentage removal of Hg(II) has increased with regards to an increase in the mass of sorbents. This is due to the fact that the increased dosage of MIP eventually increased the binding sites that are available for the sorption of Hg(II) onto the MIP’s surface [20].

### 2.4. Adsorption Isotherm

**Figure 6:**
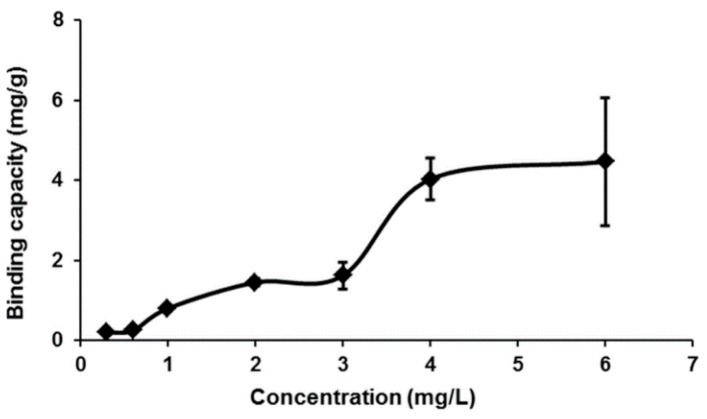
Effect of equilibrium Hg(II) concentration on the adsorption of Hg(II) ions on MIP (conditions: total volume of 10 mL, 10 mg of MIP beads, pH 7, temperature 25 °C, contact time 10 s).

Equilibrium data, also known as the adsorption isotherm, are the basic requirement for designing an adsorption system. The sorption isotherm was measured for Hg(II) using MIP by varying the initial concentration of Hg(II) solution, and the results are shown in Figure 6. From the analysis of the results, a significant sorption capacity was observed at 4.46 mg/g. In this work, the equilibrium data for Hg(II) on MIP was modeled with the Langmuir and Freundlich model [21].

The Langmuir model may be represented in a linear equation form as below:

(1) $$\frac{C_{e}}{q_{e}}=\frac{C_{e}}{Q_{m}}+\;\frac{1}{{bQ}_{m}}$$

where *C_e_* is the equilibrium concentration (mg/L), *q_e_* is the amount of Hg(II) adsorbed at equilibrium (mg/g) and *Q_m_* and *b* are Langmuir constants, which are related to the sorption capacity and energy of sorption, respectively. The plot of *C_e_*/*q_e_* against *C_e_* was used to validate the Langmuir isotherm.

The Freundlich isotherm is an exponential equation and therefore assumes that as the sorbate concentration increases, the concentration of sorbate on the adsorbent surface also gets increased. The frequently used linear equation for the Freundlich isotherm is shown below:

(2) $$\log q_{e}\text{=}\log K_{f}\text{+}\frac{1}{n}\log C_{e}$$

where *K*_f_ is the intercept showing the sorption capacity of the sorbents and 1/n is the slope showing the variation of the sorption with concentration.

**Figure 7:**
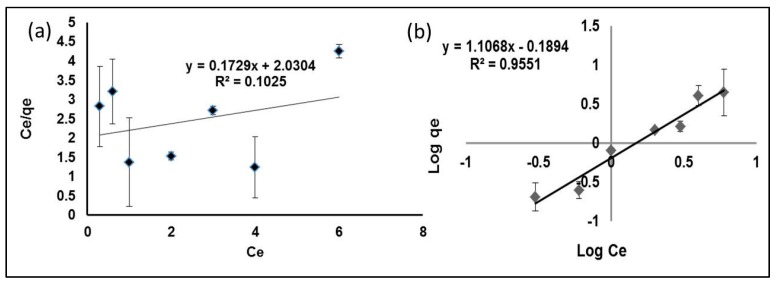
Langmuir (**a**) and Freundlich (**b**) plots for the sorption of Hg(II) by MIP.

Both Langmuir and Freundlich plots for the sorption of Hg(II) are shown in Figure 7a,b, and the related constants are given in Table 1. From the results, the value of the correlation coefficient for the Freundlich plot is high (*R*^2^ = 0.9551) compared with the Langmuir plot (*R*^2^ = 0.1025). The calculated value of *K*_f_ for the Freundlich model is comparable to the experimental value *q*_e_, and thus, it can be concluded that the sorption of Hg(II) onto MIP obeys the Freundlich model. The Freundlich isotherm gives the relationship of equilibrium between the liquid and solid phases based on the multilayer adsorption (heterogeneous surface). The Freundlich isotherm explains this better, probably due to the multiple interaction and adsorption that occurs on multiple layers of the adsorbates. Besides this, the Freundlich model has been shown to be generally applicable to most of the non-covalently-imprinted polymers [22,23].

**Table 1:** Langmuir and Freundlich adsorption constants for the MIP beads.

Experimental (*q*_e_ = mg/g)	Langmuir Constant			Freundlich Constant		
	*Q*_m_ (mg/g)	*b*_L_ (L/mg)	*R*^2^	*K*_f_ (mg/g)	1/n (L/mg)	*R*^2^
4.46	5.7837	0.0505	0.1025	0.6465	1.1068	0.9551

### 2.5. Adsorption Kinetics

**Figure 8:**
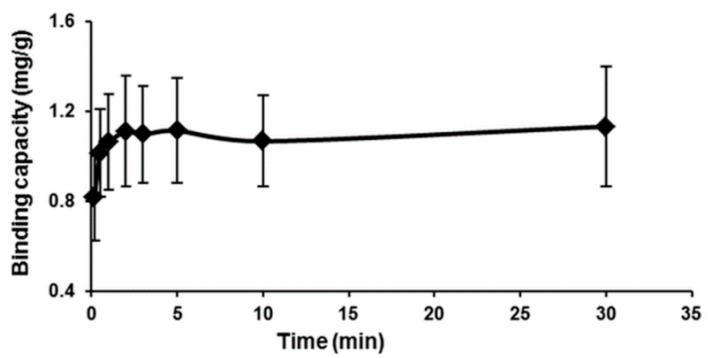
Time-dependent adsorption of Hg(II) ions on the MIP beads (conditions: 50 mL of 2 mg/L Hg(II) ions solution, 50 mg polymer, at pH 7 and 25 °C).

To determine the rate of loading of Hg(II) on the MIP, the binding capacity as a function of time was measured and is shown in Figure 8. From the figure, the Hg(II) sorption process seems to be rapid in the first few seconds and rather slow while approaching an equilibrium stage. The maximum adsorption capacity occurred over a 30-min period investigated to be around 1.132 mg/g, which is equivalent to about an 89.0% sorption,* i.e.*, confirming the higher sorption capacity in a short period of time.

The sorption kinetic data of Hg(II) were analyzed using Langergen’s first order rate model, and the equation involved is shown below [24]:

(3) $$\text{ln }(q_{\text{e}}-q_{\text{t}}\text{) }=\text{ ln }\text{(}q_{\text{e}}\text{) }\text{−}K_{\text{1}}t$$

where *K*_1_ (min^−1^) is the rate constant of pseudo-first order sorption, *q*_t_ denotes the amount of Hg(II) sorption (mg/g) at times t (min) and *q*_e_ denotes the amount of Hg(II) sorption (mg/g) at equilibrium.

In addition, the sorption kinetic data for Hg(II) was also analyzed with the pseudo-second order equation based on the adsorption equilibrium capacity, as shown below [25]:

(4) $$\frac{t}{q_{\text{t}}}=\frac{1}{K_{2}q_{\text{e}}^{2}}+\frac{t}{q_{\text{e}}}$$

The plot *t*/*q*_t_* versus* t should give a straight line if the second order kinetics is applicable, and the values *q*_e_ and *K*_2_ can be calculated from the slope and the intercept of the plot, respectively. This model is more likely to predict the kinetic behavior of sorption, with chemical sorption being the rate-controlling step [26].

**Figure 9:**
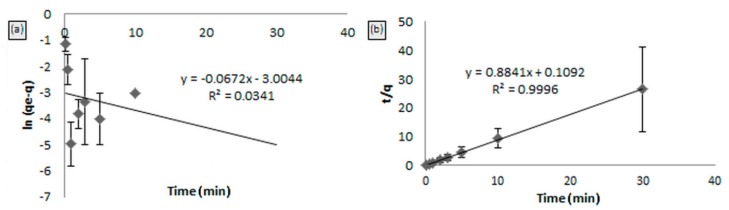
Kinetic model: (**a**) pseudo-first order kinetic model and (**b**) pseudo-second order kinetic model for MIP beads.

The pseudo-first and second order kinetics towards the adsorption of Hg(II) on MIP are shown in Figure 9a,b. From the figure, the linear plot of ln (*q*_e_* − q*)* versus* *t* for the pseudo-first order kinetics (Figure 9a) shows a low correlation coefficient (*R*^2^ = 0.0341). Moreover, Table 2 shows a large difference of experimental and calculated sorption capacities indicated for a poor pseudo-first order fit to the experimental data. However, from Figure 9b, the pseudo-second order model was likely a straight line having a high correlation coefficient (*R*^2^ = 0.999). The values of rate constants summarized in Table 2 provide the information that the theoretical value of *q*_e_ (cal) was closer to the experimental *q*_e_ (exp) value for the pseudo-second order. From the analysis of the results, it is evident, therefore, that the pseudo-second order kinetic model provided a good correlation for the sorption of Hg(II) onto MIP [17].

**Table 2:** The first and second order kinetic constants for the MIP beads.

Experimental *q*_e_ (exp)	First Order			Second Order		
	*K*_1_ (min^−1^)	*q*_e_ (calc) (mg/g)	*R*^2^	*K*_2_ (mg/g·min^−1^)	*q*_e_ (calc) (mg/g)	*R*^2^
1.132	0.0672	0.0496	0.0341	7.1577	1.074	0.9996

### 2.6. Selectivity Studies

The competitive sorption of Hg(II)/Zn(II), Hg(II)/Cd(II) and Hg(II)/Pb(II) from the mixtures of all respective metals was conducted using MIP and NIP. Zn(II) and Cd(II) were chosen as competitive metals for Hg(II), because of their similar atomic radii, while Pb(II) was chosen because of a similar period number, which means that they both have six occupied energy shells. Further, the distribution and selectivity coefficient of Hg(II) with respect to other metals were calculated using the equation shown below [27].

(5) $$K_{\text{d}}\text{ = }\frac{(C_{i}-C_{f})V}{M}$$

where *K*_d_, *C*_i_ and *C*_f_ represent the distribution coefficient and initial and final solution concentrations (mg/L), respectively. *V* is the volume of the solution (L), and *M* is the mass of the sorbents (g). The selectivity coefficient of the binding of an ion with the competitor ions can be obtained from the equilibrium data according to Equation (7).

(6) $$K=\frac{K_{d}\text{ (template metal)}}{K_{d}\text{ (interferent metal)}}$$

where *K* is the selectivity coefficient of interfering metals. A comparison of the *K* value of the imprinted polymer with those metal ions allows an estimation of the effect of imprinting on selectivity. In order to evaluate the imprinting effect, a relative selectivity coefficient *K' *was defined as follows:

(7) $$K^{\prime }=\frac{K\text{(imprinted)}}{K\text{(non-imprinted)}}$$

Table 3 summarizes the distribution coefficient (*K*_d_), selectivity coefficient *k* values of Cd(II), Zn(II) and Pb(II) with respect to Hg(II). From the table, the comparison of *K*_d_ values for Hg(II) with other competitive metals shows a high *K*_d_ value, while showing decreasing values for Zn(II), Cd(II) and Pb(II). The relative selectivity coefficient *K'* is an indicator for expressing the metal adsorption affinity of recognition sites to the imprinted Hg(II) ions [17]. These results show that the relative *K'* values for the Hg(II)/Zn(II), Hg(II)/Cd(II) and Hg(II)/Pb(II) are 2.03, 1.50 and 1.46, respectively,* i.e.*, all of the values are higher than one. This proves that the presence of Hg(II) can be determined even with the existence of Zn(II), Cd(II) and Pb(II), which further provides evidence for the promising application of our synthesized MIP for the separation of Hg(II) ions.

**Table 3:** *K*_d_, *K*, and *K'* values of Zn(II), Cd(II) and Pb(II) with respect to Hg(II).

Metal Ion	MIP		NIP		*K'*
	*K*_d_	*K*	*K*_d_	*K*	
Hg(II)	0.87	-	0.66	-	-
Zn(II)	0.43	2.05	0.66	1.00	2.03
Cd(II)	0.54	1.60	0.62	1.07	1.50
Pb(II)	0.04	22.52	0.04	15.42	1.46

### 2.7. Sorption of Hg(II) from Petroleum Oil

**Figure 10:**
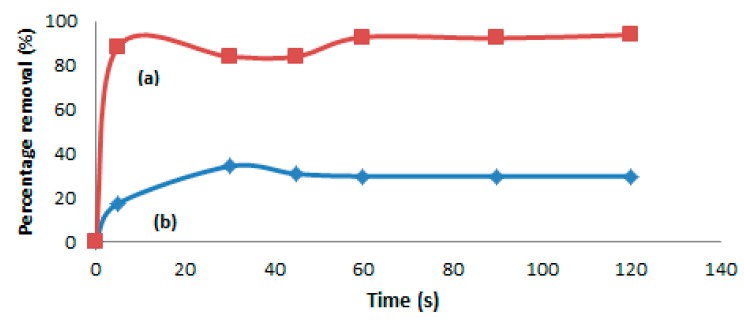
Comparison of the sorption capacities for (a) sludge and (b) crude oil samples.

The sorption of Hg(II) was carried out in real samples, which are sludge and crude petroleum oil. The samples were diluted with petroleum ether with 1:5 for crude oil samples and 1:15 for sludge sample. The MIP sorbents were treated with 2.0 mL methanol and 2.0 mL water before spiking with real samples. The samples were analyzed by a mercury analyzer, Nippon brand (Series Number NIC SP3D), and were done in HG solution Sdn. Bhd., Paka (Terengganu, Malaysia). Further, the samples were treated by using 10 mL of sample solutions in 10 mg of MIP beads at room temperature. Figure 10a shows that the percentage removal of Hg(II) in the sludge sample getting increased rapid up to 93.8% within the first 2 min of treatment by the MIP particles. This fast absorption of MIP beads towards mercury ions is probably due to the higher electrostatic and geometric shape affinities between the Hg(II) ions and Hg ion cavities with those of the lone pair groups in the MIP microstructure. Similarly, during the removal of Hg(II) from the crude oil sample, the percentage removal of Hg(II) also seems to be increased from 17.4% (within 5 s) to 29.8% after 2 min, as indicated by the graph shown in Figure 10b. It can also be seen from Figure 10a,b that the adsorption capacities are increasing with time, and the Hg(II) adsorption is fast during the first few seconds, until it reaches an equilibrium level that is achieved after 60 s for the sludge and 30 s for the crude oil sample. This represents the saturation of active binding cavities on the MIP particles, further confirming the potential usage of the synthesized MIP for the removal of Hg or its ions from the industrial samples.

Currently, the different approaches available for mercury removal include gas stripping, chemical precipitation, chemical adsorption and reverse osmosis. The gas stripping and reverse osmosis methods are relatively expensive, require heavy machinery and cannot be applied for pilot projects. The chemical precipitation, though, is a simple and easy method, but the use of toxic compounds, such as H_2_SO_4_, Na_2_S_2_O_3_ or I_2_ limits its use for all samples on an industrial scale [8]. To overcome these limitations, we have followed the chemical adsorption technique for the selective removal of Hg(II) ions or its compounds by taking advantage of the lone pair electron groups of cysteine and thereby forming an MIP complex. Thus, the developed complex was found to be highly stable at elevated temperature conditions, as indicated by the thermal studies and further proved its efficiency by adsorbing the Hg(II) ions of 37.26 µg/g at a neutral pH of 7. This property of the higher adsorption efficiency by the MIP at neutral pH is of utmost importance, as no other harsh conditions (acidic, basic or organic solvents) have to be maintained, and the reaction can be carried out with simple water (green approach), unlike other methods. Further, the selectivity efficiency of the MIP towards Hg(II) was confirmed by the presence of other competitive metal ions, such as Cd(II), Zn(II) and Pb(II). The testing of the material with other industrial samples isolated about 94% of Hg(II) from sludge and 30% from the crude oil samples during a fast time interval of 2 min, confirming again its applicability for real-time analysis. We believe that our proposed method is superior compared to other mercury removal methods of gas stripping, reverse osmosis and chemical precipitation, where expensive handling, complex equipment and toxic solvents have to be used.

## 3. Experimental Section

### 3.1. Materials

Methacrylic acid was purchased from Fluka (Steinheim, Germany). 2-hydroxyethyl methacrylate (HEMA), ethylene glycol dimethacrylate (EGDMA), (methacryloyl oxyethyl trimethyl) ammonium chloride, and l-cysteine were obtained from Sigma-Aldrich (St. Louis, MO, USA), stored at 4 °C until use. Benzoyl peroxide was obtained from R & M chemical (Essex, UK). All other chemicals and solvents used were of analytical grade and were used as received without any further purification.

### 3.2. Preparation of Cysteine Complex

The synthesis procedure used for the complexation of the cysteine ligand was adapted from elsewhere, but a small change of chemicals was applied [1]. Briefly, about 5 g of cysteine ligand and 0.2 g of sodium nitrate (NaNO_2_) were dissolved in 30 mL of K_2_CO_3_ aqueous solution (5% *v*/*v*) and then cooled to 0 °C. To this solution, 4 mL of (methacryloyl oxyethyl) trimethyl ammonium chloride were added slowly under a nitrogen atmosphere. The resulting solution was magnetically stirred at room temperature for about 2 h; at the end of this period, the pH of the solution was adjusted to 7.0 and further extracted by ethyl acetate. The aqueous phase was evaporated in a rotary evaporator and the residue (cysteine complex) was crystallized by using ethanol and ethyl acetate solvents.

### 3.3. Preparation of Cysteine Complex-Hg-Imprinted and Non-Imprinted Polymer

The Hg-imprinted polymer was synthesized using the thermally-induced free radical polymerization method. For that, solid cysteine-complex ligand (2.0 mmol) was added slowly to 50 mL of methanol and then treated with mercury nitrate, Hg(NO_3_)_2_ (1.0 mmol), solution at room temperature. The mixture was stirred for 20 min before 5 mL of this solution were pipetted into a borosilicate bottle, and MAA (6.0 mmol), HEMA (3.0 mmol), EGDMA (30 mmol) and 0.1 g benzoyl peroxide were added. The mixture was stirred for 30 min, followed by purging with nitrogen gas for 10 min before being sealed. Then, the mixture was transferred into a 70 °C water bath for polymerization, and the process was continued for 2 h. After formation, the polymer was broken into large pieces before being crushed into small particles using a mortar and pestle. The MIP particles were washed with methanol/water solution (60/40, *v*/*v*) for 24 h, 0.5% thiourea in 0.05 M HCl solution for 48 h at room temperature and cleaned with 0.1 M HNO_3_ in a magnetic stirrer for 3 h. The non-imprinted polymer (NIP) was synthesized in a similar way, but in the absence of the template molecule, Hg(NO_3_)_2_.

### 3.4. Characterization of MIP and NIP

For the characterization of MIP and NIP, various instruments were used, which included the FTIR Perkin Elmer 1600 Spectrophotometer (Foster City, CA, USA) for the Fourier transform infrared (FTIR) spectroscopy, JEOL field emission scanning electron microscopy (FESEM) from JEM 1200 EX (Tokyo, Japan) for the surface morphology and an inductively-coupled plasma-mass spectrometer (ICP-MS) from Perkin Elmer Sciex ELAN DRC-e for the elemental analysis.

### 3.5. Adsorption Studies

The solid phase extraction (SPE) method was applied in order to study the adsorption of Hg(II) ions from aqueous solutions against the effects of the pH, dosage, adsorption isotherm, kinetics and selectivity of the fabricated MIP and NIP. For that, the MIP and NIP particles were placed above the polyethylene frit inside the empty SPE cartridge and conditioned with 2 mL each of methanol and deionized water. Following the conditioning, the Hg(II) solution was allowed to pass through the cartridge at a flow rate of 0.25 mL/min, and the samples were collected after the completion of passing. The initial and final concentrations of Hg(II) were determined using the ICP-MS analysis, and the sorption capacity was calculated using Equation (8) shown below:

(8) $$q=\frac{(C_{o}-C_{e})\times V}{M}$$

where *q* (mg/g) is the amount of the total adsorption of Hg(II) and *C*_o_ and *C*_e_ are the initial and equilibrium concentrations of Hg(II) in solution (mg/L), respectively. *V* (L) is the volume of the solution, and *M* (g) is the weight of MIP.

The effect of the dosage of MIP and NIP particles on the sorption of Hg(II) was studied by varying the amounts of MIP and NIP (5, 10, 30, 50, 70 and 100 mg), while maintaining the concentration and volume of Hg(II) solution, 10.0 mL of 200 µL/L Hg(II) of 1000 ppm stock solution. The sorption isotherm was measured by using 10 mg of MIP particles in 10 mL of Hg(II) solution with different concentrations (0.3, 0.6, 1.0, 2.0, 3.0, 4.0 and 6.0 mg/L). The adsorption kinetics were studied by using 50 mg of MIP and 50 mL of Hg(II) solution. The pH was adjusted to an optimum pH, and the Hg(II) samples were collected at various time intervals (0.16, 1, 2, 3, 5, 10 and 30 min).

The selectivity of MIP and NIP was determined by studying the sorption of MIP and NIP towards Hg(II) ions against the competitive ions, that include Pb(II), Cd(II) and Zn(II). A solution of 10 mL containing 10 mL/L of Hg(II) and the competitor ions were mixed together and treated with MIP and NIP (10 mg) at room temperature. The concentrations of the metal ions were measured by ICP-MS, and the binding capacity and distribution coefficient were calculated.

Similarly, the desorption of Hg(II) ions was studied by using 0.5% thiourea in 0.05 M HCl solution. Then, the MIP was dried and reused for the sorption of Hg(II). The Hg(II) ions’ sorption-desorption procedure was repeated for 4 times using the same MIP inside the cartridge.

### 3.6. Adsorption of Hg(II) Ions from Petroleum Oil Samples

The sorption of Hg(II) ions on MIP from petroleum oil was studied by using the same SPE method. For that, the derivation of petroleum oil, that is the crude and sludge samples, was obtained from HG solution Sdn. Bhd, Malaysia.

### 3.7. Statistical Analysis

Data are presented as the mean ± SD of 3 separate experiments, and the statistical analyses were performed using a one-way analysis of variance (ANOVA) method for multiple comparisons. A probability of *p <* 0.05 was considered statistically significant and *p < *0.01 as highly statistical significant.

## 4. Conclusions

In this study, we developed a cysteine-complexed MIP to serve as a useful technique for the removal of heavy metal ions by using electrostatic and van der Waals chemistry between lone pair electron groups of cysteine and Hg(II) ions. The MIP of the cysteine complex prepared by the bulk polymerization method was thoroughly characterized by FTIR, FESEM and TGA for the bonding, morphology and thermal stability, respectively. The studies of the effect of pH, dosage, adsorption isotherm, adsorption kinetics and selectivity parameters provide the information that our cysteine-complexed MIP is functioning effectively for the trapping of Hg(II) ions at a neutral pH. Further, the sorption isotherm studies indicated that the Freundlich isotherm fits well for the adsorption of Hg(II) onto the MIP and is investigated to follow the pseudo-second order kinetics. Finally, our cysteine-complexed MIP also proved its selectivity efficiency towards Hg(II) ions when other competitive metal ions, such as Cd(II), Zn(II) and Pb(II), were present in the samples. In addition, its applicability for the real-time analysis of samples was confirmed when 94% of Hg(II) from sludge and 30% from crude oil samples were observed within a 2-min time interval. All of these results therefore confirm the promising behavior of our newly synthesized cysteine-complexed MIP towards the separation of toxic pollutants from crude and petroleum oils and other industrial wastes.
